# The Impact of CKD Anaemia on Patients: Incidence, Risk Factors, and Clinical Outcomes—A Systematic Literature Review

**DOI:** 10.1155/2020/7692376

**Published:** 2020-07-01

**Authors:** Eirini Palaka, Susan Grandy, Heleen van Haalen, Phil McEwan, Oliver Darlington

**Affiliations:** ^1^Global Payer Evidence, AstraZeneca, Cambridge, UK; ^2^Global Payer Evidence, AstraZeneca, Gaithersburg, USA; ^3^Global Payer Evidence, AstraZeneca, Gothenburg, Sweden; ^4^Health Economics and Outcomes Research Ltd, Cardiff, UK

## Abstract

Anaemia is a common consequence of chronic kidney disease (CKD); however, the risk factors for its development and its impact on outcomes have not been well synthesised. Therefore, we undertook a systematic review to fully characterise the risk factors associated with the presence of anaemia in patients with CKD and a contemporary synthesis of the risks of adverse outcomes in patients with CKD and anaemia. We searched MEDLINE, EMBASE, and the Cochrane Library from 2002 until 2018 for studies reporting the incidence or prevalence of anaemia and associated risk factors and/or associations between haemoglobin (Hb) or anaemia and mortality, major adverse cardiac events (MACE), hospitalisation, or CKD progression in adult patients with CKD. Extracted data were summarised as risk factors related to the incidence or prevalence of anaemia or the risk (hazard ratio (HR)) of outcome by Hb level (<10, 10–12, >12 g/dL) in patients not on dialysis and in those receiving dialysis. 191 studies met the predefined inclusion criteria. The risk factor most associated with the prevalence of anaemia was CKD stage, followed by age and sex. Mean HRs (95% CI) for all-cause mortality in patients with CKD on dialysis with Hb <10, 10–12, and >12 g/dL were 1.56 (1.43–1.71), 1.17 (1.09–1.26), and 0.91 (0.87–0.96), respectively. Similar patterns were observed for nondialysis patients and for the risks of hospitalisation, MACE, and CKD progression. This is the first known systematic review to quantify the risk of adverse clinical outcomes based on Hb level in patients with CKD. Anaemia was consistently associated with greater mortality, hospitalisation, MACE, and CKD progression in patients with CKD, and risk increased with anaemia severity. Effective treatments that not only treat the anaemia but also reduce the risk of adverse clinical outcomes are essential to help reduce the burden of anaemia and its management in CKD.

## 1. Introduction

The worldwide prevalence of chronic kidney disease (CKD) is estimated to be 8–16% [1] and continues to grow, driven by ageing populations and increasing rates of obesity and type 2 diabetes mellitus. Type 2 diabetes and hypertension are the leading causes of CKD in the developed world [1], and the presence of CKD increases san individual's risk of developing cardiovascular (CV) disease, hyperlipidaemia, mineral and bone disorders, and anaemia [2]. Anaemia—defined by the World Health Organization (WHO) as a blood Hb concentration of <12 g/dL for nonpregnant adult women and <13 g/dL for adult men [3]—is characterised by insufficient red blood cells and therefore haemoglobin (Hb), and common signs/symptoms include a pale appearance, fatigue, and dyspnoea (breathlessness).

The development of anaemia in patients with CKD is driven by at least two factors. First, compared with patients without CKD, those with CKD produce less erythropoietin (EPO), a hormone produced by the kidneys that stimulates red blood cell production [4], and second, hepcidin—a hormone that (at high levels) impairs dietary iron absorption—is elevated in patients with CKD [5]. Iron is an essential component of Hb and is therefore necessary for oxygen transport. Whilst it is well established that that the presence of anaemia in patients with CKD is associated with poorer quality of life [6] and increased risks of adverse clinical outcomes [2], no known systematic literature reviews (SLRs) or meta-analyses have quantified the risk of adverse clinical outcomes against the severity of anaemia. Therefore, we sought to review the literature to better understand the risk factors associated with the prevalence of anaemia in patients with CKD treated according to standard of care and to quantify the associations between anaemia (or Hb concentration) and the risks of patient mortality, hospitalisation, major adverse cardiac events (MACE), or CKD progression.

## 2. Methods

### 2.1. Literature Search and Data Extraction

A SLR was conducted according to the Preferred Reporting Items for Systematic review and Meta-Analysis Protocols (PRISMA-P) checklist [7]. Searches were conducted to include studies published between 01 January 2002 and 31 August 2018 across the following electronic databases: PubMed (MEDLINE and MEDLINE In-Process), EMBASE, and the Cochrane Library. The electronic search strategies for each of the databases are included in the Supplementary Material (available here). In addition to the searching of databases and conference proceedings, a free text Internet search was conducted and reference lists from relevant studies were used to identify further studies that may meet eligibility criteria. Search strategies for each database are detailed in Supplementary Tables S1–S3.

Bibliographic details and abstracts of all citations retrieved by the literature search were downloaded into Endnote version X7. Titles and abstracts were independently assessed for eligibility by two reviewers. Full texts of potentially eligible studies were retrieved and assessed against the Population-Intervention-Comparators-Outcomes-Study (PICOS) eligibility criteria (Table 1) independently by two reviewers. Any discrepancies between the two reviewers concerning eligibility were resolved by consensus.

The inclusion criteria captured studies describing the incidence or prevalence of anaemia alongside associated risk factors and/or associations between Hb or anaemia and mortality, MACE, hospitalisation, or CKD progression in adult patients with CKD on dialysis or adult patients with CKD not on dialysis. “Associations” included all studies that reported relative or absolute measures of risk for mortality, hospitalisation, MACE, and CKD progression dependent on a patient's Hb level or the presence of anaemia and were reported in terms of event incidence rate, probability of event, hazard ratio (HR), odds ratio (OR), relative risk ratio (RR), and incidence rate ratios (IRRs), irrespective of significance. Most studies reported measures of risk adjusted for baseline demographics and comorbidities, and the most adjusted measure was extracted for analysis. MACE included cardiovascular events, stroke, coronary heart (or artery) disease, heart failure, myocardial infarction, and atrial fibrillation. Studies conducted in paediatric patients or those reported in languages other than English were excluded. Data from studies that met the inclusion criteria were extracted by a single reviewer and quality-checked by a second reviewer. Due to wide heterogeneity in study design across the included studies, a quality assessment was not performed.

### 2.2. Analysis

Extracted data were tabulated to identify frequently reported risk factors associated with the development of anaemia in patients with CKD. Risk factors were grouped into broader categories for reporting, and those identified by more than one study were displayed graphically; however, due to heterogeneity of risk factor definitions within these categories, no formal quantitative analysis is presented regarding the relative magnitude of the effects of these risk factors on the incidence of adverse clinical outcomes.

A random-effects meta-analysis of the effects of Hb concentration on the risks of all-cause mortality, CV specific mortality, MACE, hospitalisation, and CKD progression (defined as progressing to end-stage renal disease (ESRD) or the initiation of renal replacement therapy (RRT)) was conducted for studies reporting outcomes of interest in patients with CKD not on dialysis and patients with CKD receiving dialysis. Note that CKD progression was only assessed in patients with CKD not on dialysis. The meta-analysis was conducted using R 3.4.0 [8] and the package meta [9] to account for study sample sizes and reported estimates of uncertainty.

To enable more consistent analysis of reported associations between Hb concentration and patient adverse outcomes (i.e., mortality, MACE, hospitalisation, and CKD progression), Hb concentration categories were converted to a single Hb value for analysis using the midpoint of the categorical range. Hb was grouped into the following categories for analysis: <10 g/dL, 10–12 g/dL, and >12 g/dL, and risk of each outcome attributed to each Hb grouping where data were available. If a study reported Hb without a defined lower limit (e.g., <9 g/dL), the upper defined limit minus 1 g/dL (i.e., 8 g/dL) was applied to that category prior to inclusion into the overall analysis. Similarly, if a study reported Hb without a defined upper limit (e.g., >13 g/dL), the lower defined limit plus 1 g/dL (i.e., 14 g/dL) was applied to that category prior to inclusion into the overall analysis. Where definitions of anaemia were not provided, a threshold value of 10 g/dL was assumed for anaemic patients and a threshold value of 13 g/dL was assumed for nonanaemic patients. Reference categories for relative measures of risk are as reported in their respective publications, and point estimates presented are based on the aggregation of relative measures of risk reported for each Hb category. Reported absolute measures of risk were converted to the corresponding relative measure for meta-analysis, using the highest Hb category as a reference.

The risk of each outcome was also expressed based on continuous Hb data (per 1 g/dL increase in Hb). Data reported are means (95% confidence interval (CI)). All reported estimates of associations between Hb or anaemia and patient outcomes were included in the analysis regardless of statistical significance.

## 3. Results

### 3.1. Summary of Included Studies

**Figure 1:**
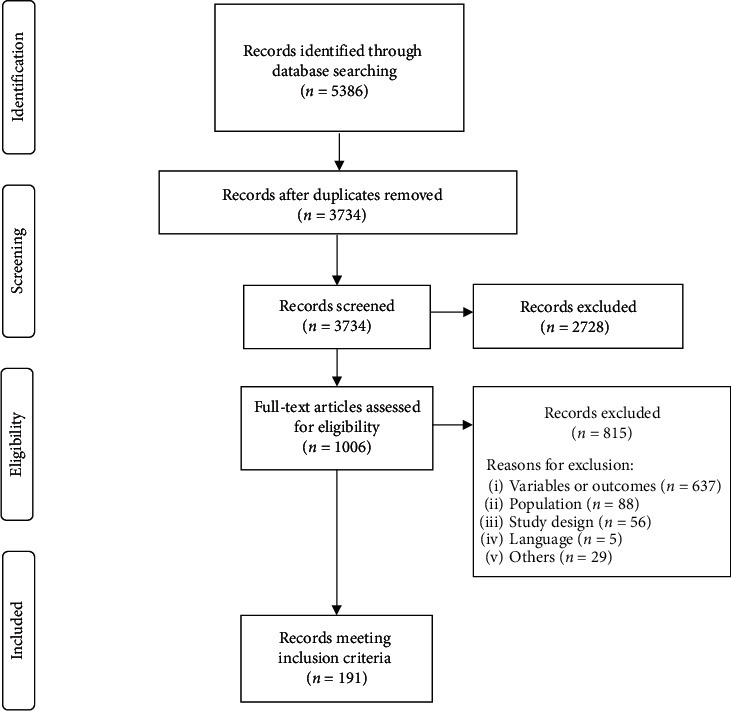
PRISMA flow diagram.

The searches identified 3734 references, after the removal of duplicates. After reviewing titles and abstracts, a further 2728 references were excluded. Full texts of the remaining 1006 references were retrieved and reviewed. Following full-text review, 191 references were deemed to satisfy the inclusion criteria and data were subsequently extracted for analysis (Figure 1). Of the 815 studies excluded following full-text review, 637 studies were excluded for not including variables (i.e., the use of haematocrit or red blood cell width distribution instead of Hb or the reporting of incidence or prevalence without associated risk factors) or outcomes (i.e., the risk of outcome was not reported in association with anaemia) of interest. Furthermore, 88 were conducted in non-CKD populations, 56 were excluded due to study design, and five were reported in languages other than English.

From the 191 included studies (Table 2), 75 were prospective, 65 were retrospective, 11 were observational, and 11 were randomised controlled trials; the design was unspecified in 29 studies. Total cohort sizes ranged from 50 to 1,136,201. Sixty-six of the included studies were conducted in Asia, 62 in North America, 48 in Europe, five in South America, four in Africa, two in Australia, and four were multinational. The majority of studies were conducted in either outpatient/renal clinic (*n* = 67) or hospital settings (*n* = 56), with small numbers undertaken in the community (*n* = 10); the setting was not reported in 58 studies.

### 3.2. Risk Factors Associated with Anaemia

**Figure 2:**
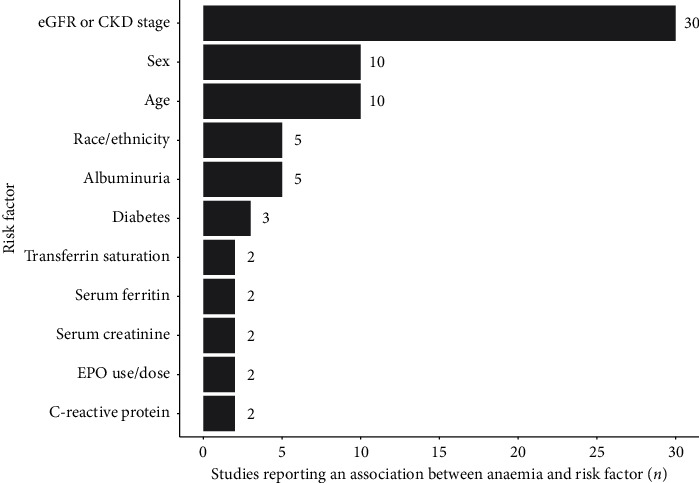
Risk factors associated with the presence of anaemia identified by more than one included study.

The incidence or prevalence of anaemia was reported in 32 studies; prevalence was reported in *n* = 30, and incidence was reported in *n* = 2. 23 studies were conducted in CKD patient cohorts not on dialysis, one study in a dialysis cohort, and eight studies in cohorts that included both nondialysis and dialysis patients with CKD. There was significant heterogeneity in the definition of anaemia used across the included studies; however, there was no trend in of the use of different Hb thresholds over time in line with changing recommendations. Whilst the most common definition of anaemia aligned with WHO guidelines for the diagnosis of anaemia [3], of Hb levels <13.0 g/dL in men and <12.0 g/dL in women (*n* = 13), thresholds ranged between 10.0 g/dL and 13.5 g/dL for men and 10.0 g/dL and 12.0 g/dL for women. Furthermore, not all studies reported separate thresholds for men and women. Risk factors associated with the presence of anaemia reported by more than one study are shown in Figure 2. The most frequently identified risk factor was eGFR or CKD stage (*n* = 30), followed by age (*n* = 10), sex (*n* = 10), race/ethnicity (*n* = 5), and albuminuria (*n* = 5). In all of these cases, patients with more severe renal impairment were at increased risk of experiencing anaemia.

### 3.3. Associations between Haemoglobin and Adverse Clinical Outcomes

#### 3.3.1. Association between Haemoglobin and Mortality

**Figure 3:**
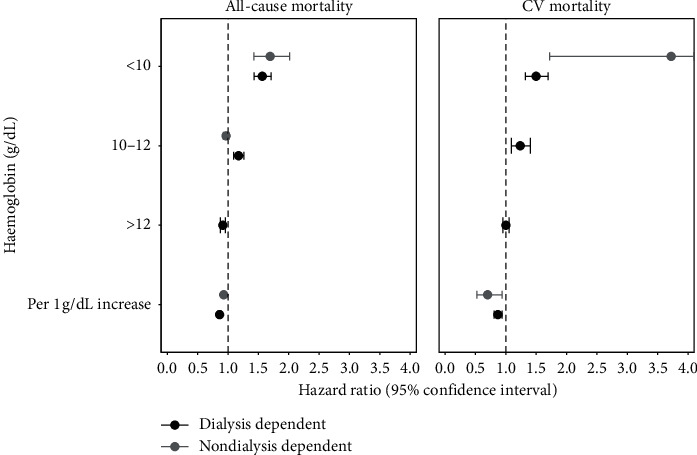
Associations between Hb level and mortality in CKD patients.

An association between Hb concentration (or anaemia) and mortality was reported by 124 studies, with 42 studies conducted in CKD patient cohorts not on dialysis, 76 studies in dialysis cohorts, and six studies in cohorts that included both nondialysis and dialysis patients with CKD. One hundred and nineteen and 13 studies identified similar relationships between Hb or anaemia and both all-cause mortality and CV mortality, respectively. Pooled mean HRs quantifying the risk of both all-cause and CV mortality based on Hb concentration are shown in Figure 3.

Pooled mean (95% CI) HRs for the risk of all-cause mortality in patients with CKD not on dialysis with Hb < 10 g/dL (*n* = 17) and 10–12 g/dL (*n* = 1) were 1.70 (1.42–2.01) and 0.97 (0.92–1.01), respectively. No studies reported the risk of all-cause mortality in patients with CKD not on dialysis with Hb > 12 g/dL. The HR of CV mortality in patients with CKD not on dialysis with Hb < 10 g/dL was only reported by one study, with a median HR of 3.72 and wide CIs of 1.72 to 8.05.

The estimated HRs of all-cause mortality in patients with CKD on dialysis with Hb < 10 g/dL (*n* = 11), 10–12 g/dL (*n* = 15), and >12 g/dL (*n* = 8) were 1.56 (1.43–1.71), 1.17 (1.09–1.26), and 0.91 (0.87–0.96), respectively. The HRs of CV mortality in dialysis patients followed a similar pattern with HRs of 1.50 (1.32–1.70), 1.24 (1.09–1.40), and 1.00 (0.95–1.06) across Hb < 10 g/dL (*n* = 4), 10–12 g/dL (*n* = 7), and >12 g/dL (*n* = 4), respectively.

The HR of all-cause mortality and CV mortality when Hb concentration was reported as a continuous variable followed similar patterns to the categorical data, of decreasing risk with increasing Hb. Mean (95% CI) HRs for the overall risk of all-cause mortality in patients with CKD not on dialysis (*n* = 18) and on dialysis (*n* = 33) were 0.93 (0.91–0.95) and 0.86 (0.83–0.89) per 1 g/dL increase in Hb, respectively. The HR of CV mortality per 1 g/dL increase in Hb was 0.70 (0.52–0.94) and 0.87 (0.81–0.94) in patients with CKD not on dialysis (*n* = 1) and on dialysis (*n* = 1), respectively.

#### 3.3.2. Association between Haemoglobin and Hospitalisation

**Figure 4:**
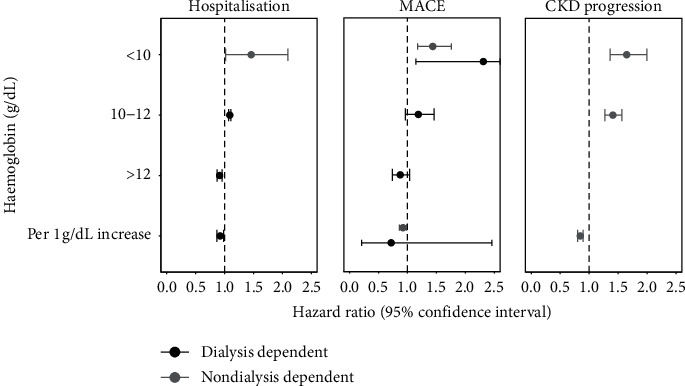
Associations between Hb level and hospitalisation, MACE, and CKD progression in CKD patients.

An association between Hb concentration (or anaemia) and hospitalisation was reported in 22 studies, with seven studies conducted in CKD patient cohorts not on dialysis, 14 studies in dialysis cohorts, and one study in a cohort that included both patients CKD not on dialysis and patients on dialysis. Mean HRs quantifying the risk of hospitalisation based on Hb concentration are shown in Figure 4 (left).

The pattern of association between Hb (or anaemia) and hospitalisation observed in the included studies tended to be similar to that observed between Hb and mortality, with lower Hb associated with higher risk of hospitalisation.

The pooled mean (95% CI) HR for the risk hospitalisation in CKD patients not on dialysis with Hb < 10 g/dL (*n* = 2) was 1.46 (1.02–2.09). No studies reported the risk of hospitalisation in CKD patients not on dialysis with Hb of 10–12 g/dL or >12 g/dL. The HR of hospitalisation in patients on dialysis with Hb 10–12 g/dL (*n* = 2) and >12 g/dL (*n* = 2) were 1.09 (1.07–1.11) and 0.91 (0.87–0.96), respectively. No studies reported the risk of all-cause mortality in CKD patients not on dialysis with Hb of 10–12 g/dL or >12 g/dL.

The overall risk of hospitalisation when Hb concentration was reported as a continuous variable followed a similar pattern to the categorical data, of decreasing risk with increasing Hb. The mean (95% CI) HR for the risk of hospitalisation in dialysis patients (*n* = 4) was 0.92 (0.87–0.98) per 1 g/dL increase in Hb. No studies reported the risk of hospitalisation in CKD patients not on dialysis where Hb was expressed as a continuous variable.

#### 3.3.3. Association between Haemoglobin and Major Adverse Cardiac Events

An association between Hb concentration (or anaemia) and MACE was reported by 30 studies. Whilst some studies reported MACE specifically, others reported single components of MACE such as stroke, heart failure, and myocardial infarction; all MACE-type events were grouped for combined analysis in this SLR. 19 were studies conducted in CKD patient cohorts not on dialysis, seven studies in dialysis cohorts, and four studies that included both CKD patients not on dialysis and patients on dialysis. Mean HRs quantifying the risk of MACE based on Hb concentration are shown in Figure 4 (middle). The risks of MACE with anaemia tended to follow a similar pattern to the risks of mortality and hospitalisations, with higher levels of risk associated with lower Hb levels.

The mean (95% CI) HR for the risk MACE in CKD patients not on dialysis with Hb < 10 g/dL (*n* = 6) was 1.44 (1.17–1.76). The estimated HRs of MACE in patients on dialysis with Hb < 10 g/dL (*n* = 1), 10–12 g/dL (*n* = 2), and >12 g/dL (*n* = 1) were 2.31 (1.14–4.66), 1.19 (0.96–1.46), and 0.88 (0.74–1.04), respectively.

The overall risk of MACE in CKD patients when Hb concentration was expressed as a continuous variable followed a similar pattern to the categorical data, of decreasing risk with increasing Hb. The mean (95% CI) HRs for MACE in CKD patients not on dialysis (*n* = 5) and on dialysis (*n* = 2) were 0.92 (0.86–0.99) and 0.72 (0.21–2.46) per 1 g/dL increase in Hb, respectively.

#### 3.3.4. Association between Haemoglobin and CKD Progression

An association between Hb or anaemia and CKD progression was reported by 38 studies. The majority (*n* = 28) defined CKD progression as progression to ESRD, with the remainder based on prespecified declines in eGFR or the doubling of serum creatinine. All studies were in cohorts of patients with CKD not on dialysis. Mean HRs quantifying the risk of CKD progression based on Hb levels are shown in Figure 4 (right).

The mean (95% CI) HRs for the risk of CKD progression in CKD patients not on dialysis with Hb < 10 g/dL (*n* = 11) and 10–12 g/dL (*n* = 1) were 1.65 (1.36–2.00) and 1.41 (1.27–1.56), respectively.

The risk of CKD progression in CKD patients not on dialysis when Hb concentration was expressed as a continuous variable followed a similar pattern to the categorical data, of decreasing risk with increasing Hb. The mean (95% CI) HR for the risk of CKD progression in CKD patients not on dialysis (*n* = 18) was 0.85 (0.80–0.89) per 1 g/dL increase in Hb.

## 4. Discussion

The aim of this review was to identify in a systematic manner the studies that reported risk factors associated with the presence of anaemia in patients with CKD and studies that characterised the association between anaemia (Hb level) and outcomes in patients with CKD. In doing so, we have summarised a contemporary evidence base of the risks of anaemia in CKD patients. In total, 191 studies that reported risk factors associated with anaemia in CKD and/or associations between Hb and mortality, hospitalisation, MACE, and CKD progression were identified. Overall, more severe anaemia was consistently associated with a greater risk of adverse outcomes.

The incidence or prevalence of anaemia in patients with CKD was reported in 31 studies identified by this systematic review. Anaemia is relatively common in CKD patients especially with increasing disease severity, with rates of up to 90% in Stage 5 patients. Studies from the UK [201] and USA [202] reported overall prevalence rates of anaemia in CKD patients with diabetes of 22% and 15%, ranging from 5% and 8% in Stage 1 up to 46% and 53% in Stage 5, respectively. Higher overall prevalence rates (32% and 52%) have been reported in studies of Malaysian [203] and Chinese [204] patients, with prevalence ranging from 13% and 22% in Stage 1 up to 70% and 90% in Stage 5, respectively.

The most commonly reported risk factor for the development of anaemia was eGFR (or CKD stage). Data consistently indicated that more severe CKD was associated with greater prevalence of anaemia. There are a number of pathophysiological mechanisms responsible for the development of anaemia alongside CKD. Compared to patients with anaemia without CKD, diseased kidneys produce less EPO than would normally be expected relative to the degree of anaemia. Whether this insufficient EPO is due to an absolute reduction in production capacity or an impaired sensitivity of kidney cells to the low tissue oxygenation that would normally stimulate production is unknown [205, 206]. More recent research has identified hepcidin as a key hormone implicated in disordered iron homeostasis in CKD patients. When elevated, hepcidin impairs dietary iron absorption and reduces the mobilisation of stored iron, further contributing to anaemia [207, 208]. Overall, anaemia in CKD is likely to be multifactorial and other factors such as shortened red blood cell survival, greater blood losses (especially in dialysis patients), and impaired absorption of dietary iron may further exacerbate the condition [4].

Older age and female sex were also commonly identified as a risk factors involved in the development of anaemia in patients with CKD. Older age is associated with greater inflammation and age-related comorbidities. The presence of proinflammatory cytokines (e.g., IL-6) increases hepcidin expression [209], likely placing older patients at higher risk of developing anaemia. Furthermore, sex hormone regulation is also impacted with older age, and both testosterone and oestrogen have been shown to reduce circulating hepcidin [210, 211]. Despite lower Hb thresholds for anaemia diagnosis in females (<12 g/dL vs. <13 g/dL for males), female patients with CKD tended to be at higher risk of developing anaemia than their male counterparts. Indeed, McClellan et al. [117] found that female patients with CKD were approximately two times more likely to develop anaemia than males. Race or ethnicity has also been identified as factors that affect the prevalence of anaemia in patients with CKD [212].

The majority of risk factors reported in the included studies were patient characteristics or comorbidities. Despite EPO and hepcidin playing a significant role in the development of anaemia in patients with CKD, only two studies identified in this SLR assessed the role of EPO as a risk factor for anaemia and no studies included hepcidin. Further work on these laboratory-based variables and their role in the risk of anaemia associated with varying levels of each biochemical parameter would be helpful to better understand the development of anaemia in patients with CKD.

Anaemia was associated with higher risks of all-cause mortality, CV mortality, MACE, hospitalisations, and CKD progression. The effects tended to increase with anaemia severity, such that a Hb < 10 g/dL was linked to comparable or higher risk of each outcome than a Hb of 10–12 g/dL. Although the effects tended to be consistent across CKD patients not on dialysis and those patients on dialysis in terms of direction, the magnitude of the risks of all-cause and CV mortality in patients with CKD not receiving dialysis appears to be greater than for those not on dialysis. The reason for this is unclear; however, due to small study numbers in some groups and an inability to control for other variables in the study populations, this finding should be interpreted with caution and may warrant further investigation. Nevertheless, the reasons for the association between anaemia and poorer outcomes are not well understood. Chronic anaemia is associated with increased cardiac output and reduced systemic vascular resistance. The low blood pressure resulting from systemic vasodilation may initiate a cascade of events, including increased sympathetic nervous activity and activation of the renin-angiotensin-aldosterone system to reduce salt and water excretion, thereby resulting in plasma volume expansion and oedema [213]. Overall, these changes result in greater cardiovascular workload, increasing the risk of conditions such as left ventricular hypertrophy [214, 215], and may contribute to the observation of higher rates of mortality, MACE, and hospitalisations observed with anaemia in CKD patients.

Anaemia is a common contributor to poor quality of life in patients with CKD but is also likely to be the factor that is most responsive to treatment [6]. Treatment of anaemia in CKD typically involves the use of supplemental iron (either oral or intravenous) and erythropoiesis-stimulating agents (ESAs) [205]. Supplemental iron may help improve iron status; however, it is not symptom- or risk-free and—depending on the mechanism(s) responsible for the anaemia—may not treat the condition adequately. ESAs mimic erythropoietin and act to stimulate red blood cell production in the bone marrow. However, although ESAs are successful at increasing haemoglobin levels in the majority of cases, they have not been shown to reduce the risks of adverse outcomes associated with anaemia in CKD patients [6] and may even result in increased risk of negative outcomes, as evidenced by three large randomised controlled trials [216–218].

The TREAT [218] study assessed the effect of darbepoetin alfa (ESA) versus placebo in more than 4000 patients with CKD, type 2 diabetes, and anaemia. HRs for death or CV event and for death or ESRD were similar; however, treatment group patients were almost two times more likely to suffer stroke (HR: 1.92 (95% CI: 1.38–2.65); *p* < 0.001) than control group patients. Additional studies have assessed the effect of ESA treatment based on different Hb target levels. The CREATE [217] trial assessed the effect of epoetin beta (ESA) administration on CV events in 603 patients with Stage 3 or 4 CKD and mild-to-moderate anaemia (Hb 11.0–12.5 g/dL). Patients were randomly assigned to receive ESA treatment to target a Hb level of 13.0–15.0 g/dL or to only receive the ESA if their Hb dropped below 10.5 g/dL (so as to maintain Hb 10.5–11.5 g/dL). ESA treatment successfully maintained Hb within predetermined ranges; however, treatment to normalise Hb (within 13.0–15.0 g/dL) was not associated with a reduction in the risk of CV events. Furthermore, the CHOIR [216] study involved the prescription of epoetin alfa (ESA) in 1400 patients with CKD to achieve a target Hb of either 13.5 g/dL (high) or 11.3 g/dL (low). Patients in the high Hb group experienced increased risk of composite (death, myocardial infarction, hospitalisation for congestive heart failure, or stroke) events (HR: 1.34 (95% CI: 1.03–1.74); *p*=0.03) and no improvement in quality of life. Post hoc analyses from this study indicate that higher ESA doses (irrespective of Hb achieved) were the primary driver for the risk of adverse outcomes [219], indicating there is a need for more effective treatments.

Based on these trials, it is clear that high Hb targets should be avoided; however, it is unclear whether the poorer outcomes are due to the ESAs themselves or factors that often accompany low Hb such as inflammation, elevated hepcidin levels, blood loss, and/or malnutrition [4]. Ultimately, this uncertainty in trying to balance the risks of untreated anaemia against the risks of treatment with ESAs has led to frustration amongst many in the clinical community. With no clear effective intervention available to both restore Hb levels and improve outcomes, Hb remains little more than a biomarker for adverse outcomes in patients with CKD.

This study is not without limitations. There was wide heterogeneity—as evidenced by I-squared statistic—in the study design, study size, patient characteristics, treatments received, outcomes (e.g., coding systems of hospitalisations), and anaemia definitions. Such variations in reporting of variables and outcomes meant that large numbers of studies were either excluded or were unable to be included in the final analysis that summarised HRs for the risk of each outcome. Furthermore, when outcome data were subdivided by Hb category and by patients receiving dialysis or not, many gaps were apparent, preventing full understanding of the level of risk. A SLR is also limited by the data available in each individual study. Whilst the most adjusted (for demographics and comorbidities) measure of risk was preferentially extracted, not all studies performed this level of analysis which may affect the overall estimate of risk.

As with all SLRs, there is also the potential for publication bias to impact on the generalisability of results. Furthermore, geographical bias may also affect the generalisability of the findings. The majority of included studies were conducted in Asia, North America, and Europe, with small study numbers in other regions. Despite this, the findings were relatively consistent irrespective of geographical location, indicating the findings are relatively generalisable across different countries and different ethnic backgrounds. The majority of studies included in this review also only presented relative risks of adverse outcomes associated with Hb or anaemia, and in general, there is a lack of information regarding the absolute risks of outcomes in these patients which may present an avenue for future research. However, the number of identified studies and the size of the cohorts represented in this review allow the formulation of an overarching summary of the associated risks to patients with anaemia.

## 5. Conclusion

This is the first known systematic review to quantify the risks of mortality, hospitalisation, MACE, and CKD progression associated with anaemia severity in patients with CKD. Anaemia was associated with greater risk of all clinical outcomes, and the risk increased with anaemia severity. The burden of CKD and its complications is substantial and projected to increase. Effective treatments that not only treat the anaemia but also reduce the risk of adverse clinical outcomes are essential to help reduce the burden of anaemia and its management in CKD.

## Figures and Tables

**Table 1:** PICOS eligibility criteria for the identification of studies.

	Inclusion criteria	Exclusion criteria
Population	Patients with CKD^*∗*^	Patients without CKD Patients with cancer Patients with MDS Paediatric patients
Intervention and comparators	NA	NA
Outcomes	Incidence or prevalence of anaemia^†^ alongside associated risk factors Anaemia or Hb concentration as a predictor of event incidence (probability, odds or rate, hazard ratio (HR), incident rate ratio (IRR), or odds ratio (OR)) for stroke, MI, heart failure, MACE, eGFR decline and/or CKD progression, progression to dialysis, death, or hospitalisation	Outcomes of interest not reported
Study	Randomised controlled trial, nonrandomised controlled trial, prospective study, longitudinal study, retrospective study, observational study, and cohort study	Economic evaluation, clinical practice or treatment guidelines, case reports, letter, editorial, and review
Language restrictions	English language only	Studies published in languages other than English
Date restrictions	2002 onwards (15-year time horizon)	Prior to 2002

CKD: chronic kidney disease; ESRD: end-stage renal disease; Hb: haemoglobin; HR: hazard ratio; IRR: incident rate ratio; MACE: major adverse cardiovascular events; MDS: myelodysplastic syndrome; MI: myocardial infarction; NA: not applicable; OR: odds ratio. ^*∗*^Patients with comorbidities were included, with the exception of CKD patients with cancer or MDS. ^†^Anaemia was defined as low Hb in the blood or low red blood cell production. Other types of anaemia such as chemotherapy-induced anaemia and sickle cell anaemia were excluded.

**Table 2:** Characteristics of the included studies reporting the incidence or prevalence of anaemia and/or associations between anaemia and risk of mortality, hospitalisation, major adverse cardiac events, or CKD progression.

Author	Year	Country	*N*	NDD (%)	DD (%)	T2DM (%)	ESA use (%)	Incidence or prevalence of anaemia?	Association between anaemia and:			
									Mortality	Hospitalisation	MACE	CKD progression
Abramson et al. [10]	2003	USA	13,716	100.0	0.0	10.6	NR	NR	N	N	Y	N
Ahmed et al. [11]	2010	USA	79,985	100.0	0.0	100.0	1.20	Prevalence	N	N	N	N
Ahn et al. [12]	2013	Korea	984	100.0	0.0	24.0	NR	Prevalence	N	N	N	N
Akizawa et al. [13]	2008	Japan	5,788	100.0	0.0	28.6	NR	Prevalence	Y	N	N	N
Akizawa et al. [14]	2014	Japan	6631	0.0	100.0	NR	100.0	NR	Y	N	N	N
Akizawa et al. [15]	2016	Japan	NR	100.0	0.0	44.5	100.0	NR	Y	N	Y	N
Anees et al. [16]	2009	Pakistan	185	0.0	100.0	NR	NR	NR	Y	N	N	N
Anees et al. [17]	2010	Pakistan	50	0.0	100.0	NR	46.0	NR	Y	N	N	N
Antunes et al. [18]	2016	Brazil	211	0.0	100.0	29.0	NR	NR	N	Y	N	N
Anutrakulchai et al. [19]	2016	Thailand	1,28,338	83.1	16.9	43.0	NR	NR	Y	Y	N	N
Arun et al. [20]	2012	India	100	NR	NR	NR	NR	Prevalence	N	N	N	N
Astor et al. [21]	2006	USA	15,792	100.0	0.0	11.6	0.0	Prevalence	Y	N	Y	N
Astor et al. [22]	2002	USA	15,419	100.0	0.0	5.0	NR	Prevalence	N	N	N	N
Avram et al. [23]	2003	USA	529	0.0	100.0	47.0	NR	NR	Y	N	N	N
Babua et al. [24]	2015	Uganda	217	100.0	0.0	16.2	NR	Prevalence	N	N	N	Y
Bae et al. [25]	2015	Korea	2,470	0.0	100.0	44.5	NR	NR	Y	N	N	N
Barrett Bowling et al. [26]	2011	USA	30,528	100.0	0.0	7.74	NR	Prevalence	N	N	N	N
Beck et al. [27]	2015	Germany	5,015	100.0	0.0	35.2	NR	NR	N	N	Y	N
Bello et al. [28]	2015	USA	4,854	100.0	0.0	100.0	0.5	NR	N	N	Y	N
Bentata et al. [29]	2013	Morocco	72	100.0	0.0	100.0	NR	NR	N	N	N	Y
Bhatti et al. [30]	2010	USA	1358	100.0	0.0	51.8	NR	NR	Y	Y	N	N
Boudville et al. [31]	2009	Multi continent	6,165	100.0	0.0	36.2	49.0	NR	Y	N	N	N
Bradbury et al. [32]	2009	USA	6,133	0.0	100.0	NR	100.0	NR	Y	N	N	N
Bravo-Jaimes et al. [33]	2015	Peru	103	0.0	100.0	NR	NR	NR	N	N	Y	N
Brunelli et al. [34]	2008	USA	34,963	0.0	100.0	45.0	NR	NR	Y	N	N	N
Brunelli et al. [35]	2008	USA	6,644	0.0	100.0	24.8	NR	NR	Y	N	N	N
Buccianti et al. [36]	2004	Italy	77	0.0	100.0	16.9	NR	NR	Y	N	N	N
Cana-Ruiu et al. [37]	2013	Romania	165	100.0	0.0	30.3	NR	NR	N	Y	N	N
Capelli et al. [38]	2008	USA	687	0.0	100.0	NR	NR	NR	Y	N	N	N
Chan et al. [39]	2009	USA	1,00,835	0.0	100.0	NR	NR	NR	N	Y	N	N
Chan et al. [40]	2017	USA	87,302	0.0	100.0	29.0	NR	NR	N	Y	N	N
Chang et al. [41]	2016	Taiwan	1,530	100.0	0.0	38.5	NR	NR	N	N	N	Y
Chen et al. [42]	2013	China	6,325	NR	NR	100.0	NR	Prevalence	N	N	N	N
Chen et al. [43]	2013	Taiwan	439	NR	NR	57.4	NR	NR	N	N	Y	N
Chen et al. [44]	2012	Taiwan	485	100.0	0.0	56.3	NR	NR	Y	N	N	N
Chen et al. [45]	2016	China	356	0.0	100.0	14.6	100.0	NR	Y	N	N	N
Chen et al. [46]	2012	China	822	100.0	0.0	20.2	NR	NR	N	N	N	Y
Chonchol et al. [47]	2008	Italy	7,389	100.0	0.0	NR	NR	Prevalence	N	N	N	N
Christensen et al. [48]	2002	USA	174	100.0	0.0	NR	NR	NR	Y	N	N	N
Conway et al. [49]	2008	USA	174	100.0	0.0	100.0	NR	NR	Y	N	Y	Y
De Nicola et al. [50]	2011	Italy	1,248	100.0	0.0	28.0	12.4	Prevalence	Y	N	N	Y
De Nicola et al. [51]	2012	Italy	1,248	100.0	0.0	27.7	12.4	NR	Y	N	N	Y
De Nicola et al. [52]	2010	Italy	668	100.0	0.0	22.6	NR	NR	Y	N	N	Y
De Nicola et al. [53]	2017	Italy	2,340	100.0	0.0	30.6	NR	NR	Y	N	N	Y
Desai et al. [54]	2011	USA	995	100.0	0.0	100.0	NR	NR	N	N	N	Y
Djukanović et al. [55]	2015	Serbia	2,153	0.0	100.0	NR	65.1	NR	Y	N	N	N
Dmitrieva et al. [56]	2013	UK	51,372	100.0	0.0	NR	NR	Prevalence	N	N	N	N
Drawz et al. [57]	2012	USA	13,874	100.0	0.0	38.8	1.8	Prevalence	N	N	N	N
Du Cheyron et al. [58]	2005	France	206	0.0	100.0	45.0	NR	NR	Y	N	N	N
Faller et al. [59]	2013	France	155	100.0	0.0	NR	NR	NR	Y	N	N	Y
Fathelrahman et al. [60]	2012	Sudan	500	0.0	100.0	NR	NR	NR	Y	Y	N	N
Feldman et al. [61]	2004	USA	27,280	0.0	100.0	44.7	93.3	NR	Y	N	N	N
Feldman et al. [62]	2002	USA	10,169	0.0	100.0	40.9	87.4	NR	Y	Y	N	N
Ferrari et al. [63]	2009	Australia	8,752	100.0	0.0	NR	NR	Prevalence	N	N	N	N
Fort et al. [64]	2010	Spain	2,310	0.0	100.0	NR	78.7	NR	Y	Y	Y	N
Frankenfield et al. [65]	2003	USA	7,723	0.0	100.0	40.5	NR	NR	Y	N	N	N
Fraser et al. [66]	2014	UK	1,707	100.0	0.0	87.6	NR	NR	Y	N	N	N
Fukuma et al. [67]	2012	Japan	95,460	0.0	100.0	32.5	100.0	NR	Y	N	N	N
Garlo et al. [68]	2015	USA	1,141	100.0	0.0	62.8	NR	NR	N	Y	N	N
Go et al. [69]	2018	USA	36,195	100.0	0.0	36.0	0.7	NR	N	N	N	Y
Goicoechea et al. [70]	2004	Spain	176	100.0	0.0	33.0	NR	NR	N	N	Y	N
Guan et al. [71]	2015	China	102	0.0	100.0	100.0	NR	NR	Y	N	N	N
Han et al. [72]	2016	Korea	17,373	100.0	0.0	9.4	NR	Prevalence	N	N	N	N
Hanafusa et al. [73]	2014	Japan	3,341	0.0	100.0	39.4	NR	NR	Y	N	N	N
Hasegawa et al. [74]	2018	Japan	2,034	100.0	0.0	38.6	NR	NR	N	N	N	Y
Hayashi et al. [75]	2013	Japan	404	0.0	100.0	41.8	58.7	NR	Y	N	N	N
He et al. [76]	2017	USA	3,557	100.0	0.0	46.3	NR	Prevalence	N	N	Y	N
Herzog et al. [77]	2004	USA	1,136,201	100.0	0.0	16.5	NR	NR	Y	N	N	N
Hosseini et al. [78]	2014	Iran	305	100.0	0.0	100.0	NR	Prevalence	N	N	N	N
Hung et al. [79]	2015	Taiwan	326	100.0	0.0	45.4	NR	NR	N	N	Y	Y
Iimori et al. [80]	2015	Japan	951	100.0	0.0	37.6	11.8	NR	Y	N	N	Y
Imamović et al. [81]	2014	Bosnia-Serbia-Slovenia	442	0.0	100.0	32.0	NR	NR	Y	N	N	N
Inker et al. [82]	2011	USA	30,528	100.0	0.0	6.50	NR	Prevalence	N	N	N	N
Inrig et al. [83]	2012	USA	1,432	100.0	0.0	31.2	100.0	NR	N	N	Y	Y
Isakov et al. [84]	2014	Israel	18,474	NR	NR	NR	NR	Prevalence	N	N	N	Y
Ishigami et al. [85]	2013	Japan	349	0.0	100.0	37.5	NR	NR	Y	N	N	N
Ishigami et al. [86]	2018	USA	5,801	100.0	0.0	15.5	NR	Prevalence	N	N	Y	N
Ito et al. [87]	2007	Japan	127	0.0	100.0	16.0	NR	NR	N	N	Y	N
Johnson et al. [88]	2007	USA	6,541	100.0	0.0	31.3	NR	NR	Y	N	N	N
Johnson et al. [89]	2008	USA	7,982	100.0	0.0	28.0	NR	NR	N	N	N	Y
Joshi et al. [90]	2014	China	805	0.0	100.0	NR	NR	NR	Y	N	N	N
Joss et al. [91]	2007	UK	508	NR	NR	NR	NR	Prevalence	Y	N	N	N
Jung et al. [92]	2015	Korea	NR	0.0	100.0	37.3	NR	NR	Y	N	N	N
Kataoka et al. [93]	2015	Japan	72	0.0	100.0	55.6	NR	NR	N	N	Y	N
Keane et al. [94]	2003	Multi continent	1,513	100.0	0.0	100.0	NR	NR	N	N	N	Y
Keane et al. [95]	2006	Multi continent	1,513	100.0	0.0	100.0	NR	NR	N	N	N	Y
Keough-Ryan et al. [96]	2005	Canada	5,549	100.0	0.0	26.2	NR	NR	Y	N	N	N
Khan et al. [97]	2017	Malaysia	621	100.0	0.0	40.1	NR	NR	N	N	N	Y
Kovesdy et al. [98]	2006	USA	861	100.0	0.0	53.1	NR	NR	Y	N	N	N
Kovesdy et al. [99]	2006	USA	853	100.0	0.0	52.8	NR	NR	Y	N	N	Y
Kovesdy et al. [100]	2006	USA	860	100.0	0.0	52.8	NR	NR	Y	N	N	N
Kuo et al. [101]	2018	Taiwan	1,558	100.0	0.0	100.0	NR	NR	Y	N	N	Y
Kuo et al. [102]	2018	Taiwan	42,230	0.0	100.0	45.4	NR	NR	Y	N	N	N
Kuwahara et al. [103]	2015	Japan	297	100.0	0.0	NR	100.0	NR	Y	N	N	N
Kwon et al. [104]	2015	Korea	1,276	0.0	100.0	52.4	71.8	NR	Y	N	N	N
Lacson et al. [105]	2009	USA	78,420	0.0	100.0	51.9	NR	NR	Y	Y	N	N
Lattanzio et al. [106]	2015	Italy	487	NR	NR	25.5	NR	NR	Y	N	N	N
Lau et al. [107]	2015	Singapore	457	100.0	0.0	64.6	0.0	Incidence	N	N	N	N
Levin et al. [108]	2008	Canada	4,231	100.0	0.0	33.0	NR	NR	Y	N	N	Y
Levin et al. [109]	2006	Canada	3,028	100.0	0.0	28.0	NR	Prevalence	Y	N	N	N
Li Vecchi et al. [110]	2007	Italy	281	0.0	0.0	64.4	NR	Prevalence	N	N	N	N
Lin et al. [111]	2004	Taiwan	105	0.0	100.0	NR	NR	NR	Y	N	N	N
Lin et al. [112]	2006	Taiwan	445	0.0	100.0	44.5	NR	NR	Y	N	N	N
Lin et al. [113]	2013	Taiwan	NR	0.0	NR	51.9	NR	NR	N	N	Y	N
Liu et al. [114]	2016	China	1,778	0.0	100.0	25.3	NR	NR	Y	N	N	N
Locatelli et al. [115]	2004	Europe	4,591	0.0	100.0	NR	NR	NR	Y	Y	N	N
MacDougall et al. [116]	2010	UK	13,422	0.0	100.0	NR	NR	NR	Y	N	N	N
McClellan et al. [117]	2004	USA	5,222	100.0	0.0	64.4	NR	Prevalence	N	N	N	N
McCullough et al. [118]	2007	USA	37,153	100.0	0.0	26.2	NR	NR	Y	N	Y	N
McMahon et al. [119]	2012	Australia	302	0.0	100.0	NR	88.4	NR	Y	N	N	N
Messa et al. [120]	2015	Italy	568	0.0	100.0	28.9	NR	NR	Y	N	N	N
Metcalfe et al. [121]	2003	UK	523	0.0	100.0	23.7	NR	NR	Y	N	N	N
Minutolo et al. [122]	2009	Italy	137	100.0	0.0	32.9	100.0	NR	Y	N	N	N
Minutolo et al. [123]	2012	Italy	194	100.0	0.0	34.0	100.0	NR	N	N	N	Y
Minutolo et al. [124]	2014	Italy	30,326	100.0	0.0	24.5	NR	Prevalence	Y	N	N	Y
Mohanram et al. [125]	2004	USA	1,468	100.0	0.0	100.0	NR	NR	N	N	N	Y
Mokoli et al. [126]	2016	Kenya	250	0.0	100.0	NR	51.6	NR	Y	N	N	N
Molnar et al. [127]	2011	USA	9,269	0.0	100.0	46.0	88.0	NR	Y	N	N	N
Moon et al. [128]	2011	Korea	250	100.0	0.0	100.0	16.0	NR	N	N	N	Y
Nakazato et al. [129]	2015	Japan	384	0.0	100.0	27.1	NR	NR	Y	N	N	N
Neves et al. [130]	2007	Portugal	95	100.0	0.0	NR	NR	NR	N	N	N	Y
Nishio et al. [131]	2013	USA	606	0.0	100.0	56.3	100.0	NR	Y	N	N	N
Nseir et al. [132]	2016	Israel	307	100.0	0.0	100.0	NR	NR	Y	Y	N	N
Ofsthun et al. [133]	2003	USA	44,550	0.0	100.0	47.9	NR	NR	Y	Y	N	N
Ohno et al. [134]	2014	Japan	2,772	100.0	0.0	0.0	NR	NR	N	N	N	Y
Okazaki et al. [135]	2014	Japan	248	0.0	100.0	48.8	100.0	NR	Y	N	N	N
Ossareh et al. [136]	2016	Iran	560	0.0	100.0	NR	NR	NR	Y	N	N	N
Panagoutsos et al. [137]	2006	Greece	299	0.0	100.0	77.9	NR	NR	Y	N	N	N
Portolés et al. [138]	2007	Spain	122	100.0	0.0	NR	NR	Prevalence	Y	Y	Y	Y
Pulliam et al. [139]	2014	USA	1,677	0.0	100.0	42.9	NR	NR	Y	Y	N	N
Rajaeefard et al. [140]	2016	Iran	290	0.0	100.0	32.4	NR	NR	Y	N	N	N
Regidor et al. [141]	2006	USA	58,058	0.0	100.0	44.7	93.0	NR	Y	N	N	N
Roberts et al. [142]	2006	USA	93,087	0.0	100.0	49.1	100.0	Incidence	Y	Y	N	N
Robinson et al. [143]	2005	USA	5,517	0.0	100.0	40.4	91.7	NR	Y	N	N	N
Rossing et al. [144]	2004	Denmark	227	NR	NR	100.0	NR	NR	Y	N	N	Y
Sabe et al. [145]	2016	USA	4,038	NR	NR	100.0	NR	NR	N	N	N	Y
Santos et al. [146]	2017	Portugal	416	100.0	0.0	50.0	NR	NR	Y	N	N	Y
Santos et al. [147]	2011	Brazil	156	0.0	100.0	NR	NR	NR	Y	N	N	N
Sato et al. [148]	2018	Japan	62,931	NR	NR	8.8	NR	NR	Y	N	N	N
Sato et al. [149]	2013	Japan	213	NR	NR	NR	NR	NR	N	N	Y	N
Sawhney et al. [150]	2009	UK and Canada	7,299	0.0	100.0	23.4	NR	NR	Y	N	N	N
Schroeder et al. [151]	2017	USA	22,460	100.0	0.0	34.3	NR	NR	N	N	N	Y
Selim et al. [152]	2007	Macedonia	214	0.0	100.0	NR	NR	NR	Y	N	N	N
Selim et al. [153]	2007	Macedonia	236	0.0	100.0	NR	NR	NR	Y	N	N	N
Servilla et al. [154]	2009	USA	12,733	0.0	100.0	47.2	97.7	NR	Y	Y	N	N
Shema-Didi et al. [155]	2010	Israel	25,800	100.0	0.0	NR	NR	NR	N	N	N	Y
Shimizu et al. [156]	2014	Japan	1,105	NR	NR	NR	NR	Prevalence	N	N	N	N
Shiraishi et al. [157]	2014	Japan	521	NR	NR	35.5	NR	NR	Y	N	N	N
Singh et al. [158]	2006	USA	1,432	100.0	0.0	50.0	100.0	NR	Y	Y	Y	Y
Skali et al. [159]	2011	USA	4,038	100.0	0.0	100.0	49.8	NR	N	N	Y	N
Song et al. [160]	2017	China	4,104	0.0	100.0	NR	NR	NR	Y	N	N	N
Spigolon et al. [161]	2016	Brazil	4,107	0.0	100.0	45.0	NR	NR	Y	N	N	N
Stevens et al. [162]	2011	USA	116,321	NR	NR	29.5	NR	Prevalence	N	N	N	N
Stevens et al. [163]	2007	UK	38,262	NR	NR	21.2	NR	Prevalence	N	N	N	N
Stevens et al. [164]	2010	Europe	1,198	100.0	0.0	100.0	NR	Prevalence	N	N	N	N
Stosovic et al. [165]	2011	Serbia	242	0.0	100.0	NR	NR	NR	Y	N	N	N
Streja et al. [166]	2008	USA	40,787	0.0	100.0	NR	NR	NR	Y	N	N	N
Sturm et al. [167]	2010	Austria	235	0.0	100.0	34.9	0.0	NR	Y	N	N	N
Szeto et al. [168]	2011	China	332	100.0	0.0	29.5	NR	NR	Y	N	N	Y
Teixeira et al. [169]	2015	Brazil	162	0.0	100.0	NR	NR	NR	Y	N	N	N
Thijssen et al. [170]	2012	USA	6,838	0.0	100.0	54.2	NR	NR	Y	N	N	N
Thorp et al. [171]	2009	USA	5,885	100.0	0.0	52.3	0.0	NR	Y	Y	N	Y
Toida et al. [172]	2017	Japan	1,375	0.0	100.0	32.4	0.0	NR	Y	N	N	N
Tripepi et al. [173]	2010	Italy	283	0.0	100.0	15.0	52.7	NR	Y	N	Y	N
Tsubakihara et al. [174]	2015	Japan	321	100.0	0.0	35.3	NR	NR	Y	N	N	N
Tsubakihara et al. [175]	2012	Japan	322	100.0	0.0	NR	NR	NR	Y	N	N	N
Ueda et al. [176]	2003	Japan	202	100.0	0.0	100.0	NR	NR	N	N	N	Y
Vaiciuniene et al. [177]	2010	Lithuania	559	0.0	100.0	NR	NR	NR	N	Y	N	N
Van Diepen et al. [178]	2014	Netherlands	394	0.0	100.0	100.0	NR	NR	Y	N	N	N
Varas et al. [179]	2018	Spain	1,679	0.0	100.0	NR	NR	NR	Y	N	N	N
Vazquez et al. [180]	2009	Spain	256	0.0	100.0	28.5	NR	NR	N	N	Y	N
Vejakama et al. [181]	2013	Thailand	1,177	0.0	100.0	21.2	NR	NR	Y	N	N	N
Voormolen et al. [182]	2010	Netherlands	547	100.0	0.0	23.6	25.1	NR	Y	N	N	N
Wagner et al. [183]	2011	Germany	215	100.0	0.0	100.0	0.0	NR	Y	N	N	N
Wagner et al. [184]	2011	UK	5,447	0.0	100.0	28.6	NR	NR	Y	N	N	N
Walker et al. [185]	2006	USA	88,657	100.0	0.0	19.6	NR	NR	N	N	Y	Y
Weiner et al. [186]	2008	USA	1,678	100.0	0.0	15.0	NR	NR	Y	N	Y	N
Weiner et al. [187]	2005	USA	2,333	100.0	0.0	17.1	NR	NR	Y	N	Y	N
Weinhandl et al. [188]	2011	USA	133,246	0.0	100.0	NR	NR	NR	Y	N	N	N
Wu et al. [189]	2013	Taiwan	1,157	100.0	0.0	0.48	NR	NR	Y	N	N	Y
Xu et al. [190]	2012	China	313	0.0	100.0	39.9	NR	NR	Y	N	N	N
Yamamoto et al. [191]	2016	Japan	2,602	100.0	0.0	27.9	6.8	NR	Y	N	N	N
Yang et al. [192]	2007	China	7,067	NR	NR	100.0	NR	NR	N	N	Y	N
Yang et al. [193]	2007	USA	34,963	0.0	100.0	45.0	NR	NR	Y	N	N	N
Yang et al. [194]	2013	China	809	0.0	100.0	23.4	NR	NR	Y	N	N	N
Yeates et al. [195]	2007	Canada	26,316	0.0	100.0	NR	NR	NR	Y	N	N	N
Yotsueda et al. [196]	2018	Japan	3,436	0.0	100.0	28.9	100.0	NR	N	N	Y	N
Zhang et al. [197]	2015	China	421	0.0	100.0	28.7	NR	NR	Y	N	N	N
Ziginskiene et al. [198]	2013	Lithuania	559	0.0	100.0	NR	NR	NR	Y	Y	N	N
Zitt et al. [199]	2014	Austria	235	0.0	100.0	34.9	77.9	NR	Y	N	N	N
Zoppini et al. [200]	2010	Italy	1,153	100.0	0.0	100.0	NR	NR	Y	N	N	N

CKD: chronic kidney disease; DD: dialysis dependent; ESA: erythropoietin-stimulating agent; MACE: major adverse cardiac events; N; no; NDD: nondialysis dependent; NR: not reported; T2DM: type 2 diabetes mellitus; Y: yes.

## Data Availability

The data supporting this systematic review are from previously reported studies and datasets, which have been cited. The processed data are available from the corresponding author on reasonable request.

## Supplementary Materials

Supplementary Materials Table S1: MEDLINE search strategy. Table S2: EMBASE search strategy. Table S3: Cochrane Library search strategy.
