# Supplementary material for: The Impact of CKD Anaemia on Patients: Incidence, Risk Factors, and Clinical Outcomes—A Systematic Literature Review
Source: Int J Nephrol. 2020 Jul 1;2020:7692376. doi: 10.1155/2020/7692376 (PMC7349626; doi:10.1155/2020/7692376)
Supplement: Supplementary Materials — Table S1: MEDLINE search strategy. Table S2: EMBASE search strategy. Table S3: Cochrane Library search strategy. [file 7692376.f1.docx]

**Supplementary Material**

**Table S1.** MEDLINE search strategy

| **#** | **Search terms** | **Number of hits** |
| --- | --- | --- |
| **1** | chronic kidney disease[MeSH Terms] OR "Chronic kidney disease"[Title/Abstract] OR CKD[Title/Abstract] OR haemodialysis[Title/Abstract] OR hemodialysis[Title/Abstract] OR dialysis[Title/Abstract] | 216,601 |
| **2** | Stroke[Title/Abstract] OR “Myocardial Infarction”[Title/Abstract] OR “Cardiovascular Disease”[Title/Abstract] OR CVD[Title/Abstract] OR “Heart Failure”[Title/Abstract] OR Mortality[Title/Abstract] OR Death[Title/Abstract] OR MACE[Title/Abstract] OR "Major adverse cardiac event"[Title/Abstract] OR Hospitalisation[Title/Abstract] OR Hospitalization[Title/Abstract] OR hospitalized[Title/Abstract] OR hospitalised[Title/Abstract] OR incidence[Title/Abstract] OR “predictive value"[Title/Abstract] OR “glomerular filtration rate”[Title/Abstract] OR eGFR[Title/Abstract] | 2,376,057 |
| **3** | anaemia[MeSH Terms] OR anaemia[Title/Abstract] OR anemia[Title/Abstract] haemoglobin[Title/Abstract] OR hemoglobin[Title/Abstract]OR “red blood cell”[Title/Abstract] OR RBC[Title/Abstract] | 155,332 |
| **4** | incidence[Title/Abstract] OR “risk factors” [Title/Abstract] OR predictor*[Title/Abstract] OR prediction[Title/Abstract] OR association[Title/Abstract] OR associations[Title/Abstract] OR risk[Title/Abstract] OR risks[Title/Abstract] OR effect[Title/Abstract] OR Score[Title/Abstract] OR effects[Title/Abstract] OR relationship[Title/Abstract] OR odds[Title/Abstract] | 8,379,867 |
| **5** | #1 AND #2 AND #3 AND #4 | 2,409 |
| **6** | Letter OR editorial OR case reports[Publication Type]) OR letter[Publication Type]) OR editorial[Publication Type]) OR review[Publication Type] | 5,525,365 |
| **7** | #5 NOT #6 | 2,096 |
| **8** | #7 Language: English | 1,971 |
| **9** | #8 AND ("2002/01/01"[Date - Publication] : "3000"[Date - Publication]) | 1,830 |

Table S2. EMBASE search strategy

| **#** | **Search terms** | **Number of hits** |
| --- | --- | --- |
| **1** | 'chronic kidney disease'/exp OR 'chronic kidney disease':ti,ab OR haemodialysis:ti,ab OR hemodialysis:ti,ab OR dialysis:ti,ab | 290,750 |
| **2** | Stroke:ti,ab OR ‘myocardial infarction’:ti,ab OR ‘cardiovascular disease’:ti,ab OR CVD:ti,ab OR ‘heart failure’:ti,ab OR mortality:ti,ab OR death:ti,ab OR mace:ti,ab OR 'major adverse cardiac event':ti,ab OR hospitalisation:ti,ab OR hospitalization:ti,ab OR hospitalized:ti,ab OR hospitalised:ti,ab OR incidence:ti,ab OR 'predictive value':ti,ab OR 'glomerular filtration rate':ti,ab OR eGFR:ti,ab | 3,325,411 |
| **3** | 'anaemia'/exp OR ‘anemia’/exp OR anemia:ti,ab OR anaemia:ti,ab OR haemoglobin:ti,ab OR hemoglobin:ti,ab OR ‘red blood cell’:ti,ab OR RBC:ti,ab | 575,483 |
| **4** | incidence:ti,ab OR 'risk factors':ti,ab OR predictor*:ti,ab OR prediction:ti,ab OR association:ti,ab OR associations:ti,ab OR risk:ti,ab OR risks:ti,ab OR effect:ti,ab OR score:ti,ab OR effects:ti,ab OR relationship:ti,ab OR odds:ti,ab | 10,719,731 |
| **5** | #1 AND #2 AND #3 AND #4 | 8,182 |
| **6** | [animals]/lim NOT [humans]/lim | 5,424,955 |
| **7** | #5 NOT #6 | 8,111 |
| **8** | #7 AND ([article]/lim OR [article in press]/lim) | 3,966 |
| **9** | #8 AND [english]/lim | 3,609 |
| **10** | #9 AND [2002-2018]/py | 3,289 |
| **11** | #10 AND [humans]/lim AND [clinical study]/lim | 2,831 |

Table S3. Cochrane Library search strategy

| **#** | **Search terms** | **Number of hits** |
| --- | --- | --- |
| **1** | MeSH descriptor: [Chronic Kidney Disease] explode all trees | 5,658 |
| **2** | (‘chronic kidney disease’ or CKD):ti,ab,kw | 8,322 |
| **3** | #1 OR #2 | 10,738 |
| **4** | (Stroke or ‘Myocardial infarction’ or ‘heart failure’ or ‘cardiovascular disease’ or CVD or Mortality or Death or MACE or ‘Major adverse cardiac event’ or Hospitalisation or Hospitalization OR Hospitalised OR Hospitalized OR Incidence OR ‘predictive value’ OR ‘glomerular filtration rate’ OR eGFR):ti,ab,kw | 260,608 |
| **5** | MeSH descriptor: [anemia] explode all trees | 4,577 |
| **6** | (anaemia OR anemia OR haemoglobin OR haemoglobin OR ‘red blood cell’ OR RBC):ti,ab,kw | 41,005 |
| **7** | #5 OR #6 | 41,299 |
| **8** | (((((((((((((incidence) or risk factors) or predictor*) or prediction) or association) or associations) or risk) or risks) or effect) or Score) or effects) or relationship) or odds) :ti,ab,kw | 806,592 |
| **9** | #3 AND #4 AND #7 AND #8 | 838 |
| **10** | ((((case report) or case study) or letter) or editorial) | 63,976 |
| **11** | letter:pt or editorial:pt or review:pt | 16,755 |
| **12** | #10 OR #11 | 71,457 |
| **13** | #9 NOT #12 | 759 |
| **14** | #13 AND publication date from Jan 2002 to present | 725 |
